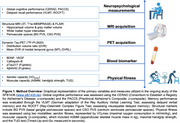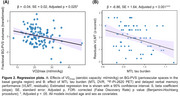# Role of physical fitness in resistance and cognitive resilience against age‐related pathology

**DOI:** 10.1002/alz70860_099453

**Published:** 2025-12-23

**Authors:** Svenja Schwarck, Niklas Behrenbruch, Beate Schumann‐Werner, Niklas Vockert, Patrick Müller, Jose Bernal Moyano, Roberto Duarte, Maria del C. Valdes Hernandez, Joanna M Wardlaw, Berta Garcia‐Garcia, Larissa Fischer, Eóin N. Molloy, Anne Hochkeppler, Enise I Incesoy, Michael Rullmann, Andrew W. Stephens, Marianne Patt, Henryk Barthel, Andreas Schildan, Barbara Morgado, Hermann Esselmann, Jens Wiltfang, Osama Sabri, Michael C. Kreissl, Emrah Düzel, Anne Maass

**Affiliations:** ^1^ German Center for Neurodegenerative Diseases (DZNE), Magdeburg, Germany; ^2^ Institute of Cognitive Neurology and Dementia Research (IKND), Otto‐von‐Guericke University, Magdeburg, Germany; ^3^ University Hospital Magdeburg, Magdeburg, Germany; ^4^ Centre for Clinical Brain Sciences, The University of Edinburgh, Edinburgh, Scotland, United Kingdom; ^5^ Division of Nuclear Medicine, Department of Radiology & Nuclear Medicine, Faculty of Medicine, Otto von Guericke University, Magdeburg, Germany; ^6^ German Center for Neurodegenerative Diseases (DZNE), Berlin, Germany; ^7^ Department of Nuclear Medicine, University of Leipzig, Leipzig, Germany; ^8^ Life Molecular Imaging GmbH, Berlin, Germany; ^9^ Leipzig University Medical Center, Leipzig, Germany; ^10^ Department of Psychiatry and Psychotherapy, University Medical Center Göttingen (UMG), Göttingen, Germany; ^11^ German Center for Neurodegenerative Diseases (DZNE), Göttingen, Germany; ^12^ Department of Psychiatry and Psychotherapy, University Medical Center Goettingen (UMG), Göttingen, Germany; ^13^ Institute of Cognitive Neurology and Dementia Research (IKND), Otto‐von‐Guericke University, Magdeburg, Sachsen Anhalt, Germany; ^14^ Faculty of Natural Sciences, Otto‐von‐Guericke University Magdeburg, Magdeburg, Germany

## Abstract

**Background:**

Cognitive reserve (CR) and brain maintenance enable the brain to maintain performance despite injury and disease while also reducing neural decline by safeguarding brain structure and function. Physical activity is a potential pathway to BM and CR, as fitness relates to better cognition in older adults, though the underlying mechanisms remain unclear. To explore the role of physical fitness in BM and CR, we tested its association with brain pathology and its potential moderation of pathology's impact on cognitive performance.

**Method:**

We collected data from 167 cognitively unimpaired participants (mean age 71.57±7.50 years; 70 females) of the ongoing SFB1436 study (www.sfb1435.de; Figure 1). We collected many markers, including global and verbal cognitive performance; aerobic (VO_2max_) and muscular capacity; blood‐based biomarkers of Alzheimer's disease (plasma Aβ1‐42/1‐40, ptau217) and plasticity (serum BDNF, VEGF and Cathepsin‐B); PET‐derived medial temporal lobe tau burden (MTL DVR, ^18^F‐PI‐2620 PET); and MRI‐derived volumes of hippocampi, white matter hyperintensities, and perivascular spaces (PVS) in the basal ganglia (BG) and centrum semiovale regions. The tests were two‐fold. We first tested whether fitness was associated with lower MTL DVR values, reduced MRI‐derived volumes of brain pathology, and better cognition. Using moderation analysis, we then tested whether physical fitness moderated the relationship between pathology and cognition. We relied on ANOVA for model comparison. We adjusted models for age and sex and FDR‐corrected multiple comparisons.

**Result:**

Participants with better aerobic capacity (VO_2max_) had lower BG‐PVS volumes (Figure 2a) and better global cognitive performance. Those with higher MTL tau burden had worse verbal memory (Figure 2b). We found no evidence of a relationship between physical fitness and Alzheimer's markers, plasticity‐related markers, or hippocampal volume. Moderation analysis revealed that physical fitness did not moderate the relationship between MTL tau burden and verbal memory, but model comparison revealed weak evidence for CR against MTL tau.

**Conclusion:**

We demonstrated that aerobic fitness is related to lower BG‐PVS volumes in old age and showed that aerobic fitness tends to act as CR proxy against MTL tau pathology. Aerobic fitness may help maintain cerebrovascular and glymphatic dysfunction in old age, thereby mitigating cognitive decline.